# Ultrasensitive Electrochemical Sensor for Luteolin Based on Zirconium Metal-Organic Framework UiO-66/Reduced Graphene Oxide Composite Modified Glass Carbon Electrode

**DOI:** 10.3390/molecules25194557

**Published:** 2020-10-05

**Authors:** Qian Wang, Chunmeng Gu, Yafen Fu, Liangliang Liu, Yixi Xie

**Affiliations:** 1 Institute of Bast Fiber Crops, Chinese Academy of Agricultural Sciences, Changsha 410205, China; q417740294@hotmail.com (Q.W.); guchunmeng02@outlook.com (C.G.); fuyafen_1995@hotmail.com (Y.F.); 2 Key Laboratory for Green Organic Synthesis and Application of Hunan Province, College of Chemistry, Xiangtan University, Xiangtan 411105, China

**Keywords:** electrochemical, glass carbon electrode, luteolin, reduced graphene oxide, UiO-66

## Abstract

Luteolin is a kind of natural flavonoid with many bioactivities purified from a variety of natural herbs, fruits and vegetables. Electrochemical sensing has become an outstanding technology for the detection of luteolin in low concentration due to its fast response, easy operation and low cost. In this study, electroreduced graphene oxide (ErGO) and UiO-66 were successively modified onto a glassy carbon electrode (UiO-66/ErGO/GCE) and applied to the detection of luteolin. A combination of UiO-66 and ErGO showed the highest promotion in the oxidation peak current for luteolin compared with those of a single component. The factors affecting the electrochemical behavior of UiO-66/ErGO/GCE were evaluated and optimized including pH, accumulation potential, accumulation time and scan rate. Under optimum conditions, UiO-66/ErGO/GCE showed satisfactory linearity (from 0.001 μM to 20 μM), reproducibility and storage stability. The detection limit of UiO-66/ErGO/GCE reached 0.75 nM of luteolin and was suitable for testing real samples. Stable detection could be provided at least eight times by one modified electrode, which guaranteed the practicability of the proposed sensor. The fabricated UiO-66/ErGO/GCE showed a rapid electrochemical response and low consumption of materials in the detection of luteolin. It also showed satisfactory accuracy for real samples with good recovery. In conclusion, the modification using MOFs and graphene materials made the electrode advanced property in electrochemical sensing of natural active compounds.

## 1. Introduction

Luteolin (3′,4′,5,7-tetrahydroxy-flavone, LU) is purified from a variety of natural herbs, fruits and vegetables and is a kind of natural flavonoid with a cis-diol structure [1]. LU has a variety of pharmacological activities and plays some important roles in the human body, such as antiallergen, antitumor, antibacterial and antivirus [2,3]. It has many medical influences on cough, inflammation, cardiovascular and other diseases [4,5]. Although LU has many good properties, high concentrations of LU can promote oxidative damage to DNA as well [6]. Based on the above reasons, it is necessary to explore a convenient and sensitive method for detecting the presence and content of LU for chemical and medical research [7]. During many years of research, various methods for detecting LU have been conducted, such as high performance liquid chromatography, spectrophotometry, gas chromatography, electrochemical methods, capillary electrophoresis, and so on [8,9,10]. In these methods, electrochemical analysis showed considerable advantages including fast response, low consumption, simple operation, accuracy and selectivity [11]. However, traditional sensors have problems in terms of linear range, electron transfer and sensitivity. Further research is still necessary because of the high medicinal value and detection requirement of LU.

Metal-organic framework materials (MOFs) are kinds of coordination polymers using inorganic metal ions as the center, and connected by external organic ligands to form a three-dimensional structure [12]. In recent years, MOFs have been widely used in the chemical field [13]. They have the characteristics of an orderly crystal structure, high porosity, and excellent thermal stability [14], which are ideal for gas storage, biosensing, catalysis, etc., [15,16]. As a kind of MOF, UiO-66 inherits the advantages of the MOFs and shows enrichment capabilities, high electroactive specific surface area, and large pores, which make it widely applicable in various fields [17]. Based on the recognition ability of LU by zirconium ions, the UiO-66 modified sensor could be utilized to detect LU. However, in order to widen the range of the detection concentration for the substance and increase the adsorption capacity of the sensor, the limited conductivity of UiO-66 should be solved.

Graphene oxide (GO) is the product of graphite powder after chemical oxidation and stripping [18]. Because the raw materials are easily available and much attention is focused on GO, it is becoming increasingly easy to obtain GO with the diverse preparation methods reported these days [19]. However, when the graphene is oxidized, the conductivity of the GO produced decreases. Accordingly, many strategies are designed for the reduction of GO and the properties of the product differ with the reducing methods. In electroreduction, GO is electrodeposited on the electrode, and then reduced by cyclic voltammetry (CV). This kind of production has high electrocatalytic activity, detection sensitivity and recognition ability [20].

**Scheme 1:**
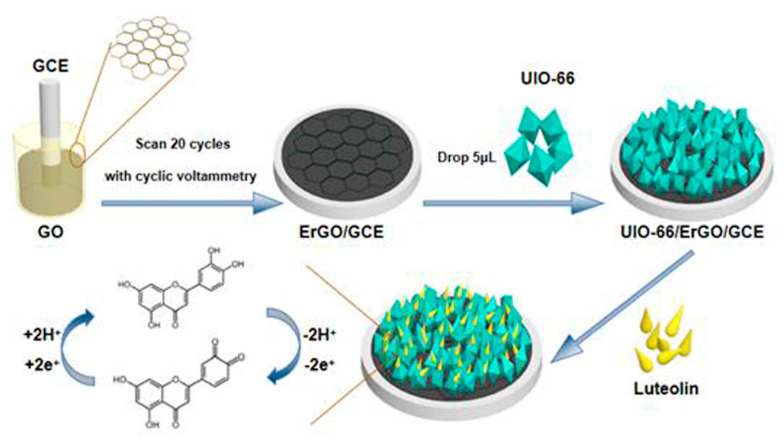
The fabrication of UiO-66/ErGO/GCE and electrochemical detection of luteolin (LU).

In this study, electroreduced graphene oxide (ErGO) and UiO-66 were successively modified onto a glassy carbon electrode (UiO-66/ErGO/GCE) and applied to the detection of LU (Scheme 1). The prepared rGO and UiO-66 were characterized by scanning electron microscope (SEM), Raman spectroscopy, Fourier transform infrared (FTIR) spectroscopy and X-ray powder diffraction (XRD) to confirm the morphology and structural properties. Many related parameters about the modified electrode were investigated and the modified electrode showed advanced properties in the electrochemical sensing of LU. The detection results for real samples with UiO-66/ErGO/GCE illustrated that it was suitable for testing real samples.

## 2. Results and Discussion

### 2.1. Characterization of UiO-66 Composites

**Figure 1:**
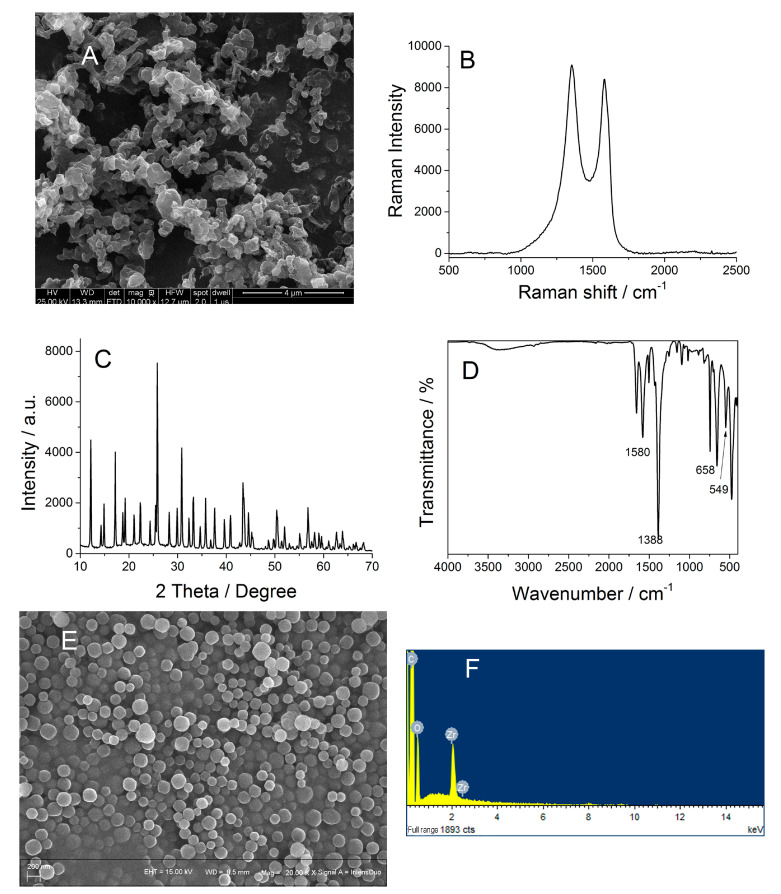
(**A**) SEM image and (**B**) Raman spectrum of rGO. (**C**) XRD pattern, (**D**) FTIR spectrum, (**E**) SEM image and (**F**) element mapping of prepared UiO-66.

The SEM image and Raman spectrum of rGO are shown in Figure 1. The small pieces and sheet-like structure, as well as the agglomeration of rGO can be found in Figure 1A. In the Raman spectrum of rGO, two apparent Raman peaks could be observed at 1352 and 1580 cm^−1^ which could be assigned as the D and G bands of rGO, respectively, (Figure 1B) [21].

To confirm the structure and morphology of prepared UiO-66, the XRD, FTIR and SEM images of UiO-66 were characterized (Figure 1C–F). As shown in Figure 1c, the typical XRD pattern of the prepared UiO-66 expressed many diffraction peaks, matching well with the reported data of simulated UiO-66 [22]. In the FTIR spectrum of UiO-66 (Figure 1D), the characteristic vibration bands at 1580 and 1388 cm^−1^ could be considered as the carboxylate asymmetric stretching and carboxylate symmetric stretching of UiO-66 [23]. In addition, the bands at 658 and 549 cm^−1^ could be assigned as Zr-O stretch vibration and Zr-(OC) asymmetric stretching, respectively. These vibrations were in accordance with reported data [24]. The SEM and elemental mapping images of prepared UiO-66 are illustrated in Figure 1E,F. It was found in the SEM image of UiO-66 that the average diameter of UiO-66 was about 200 nm with good dispersity, which was similar to that reported in the literature [25]. The elemental mapping data demonstrated that carbon atoms (81.6%, w%), oxygen atoms (13.1%) and zirconium atoms (5.3%) were observed existing in prepared UiO-66 with good distribution (Figure 1F) [26]. These characterizations showed evidence of the successful preparation of UiO-66 in this study.

**Figure 2:**
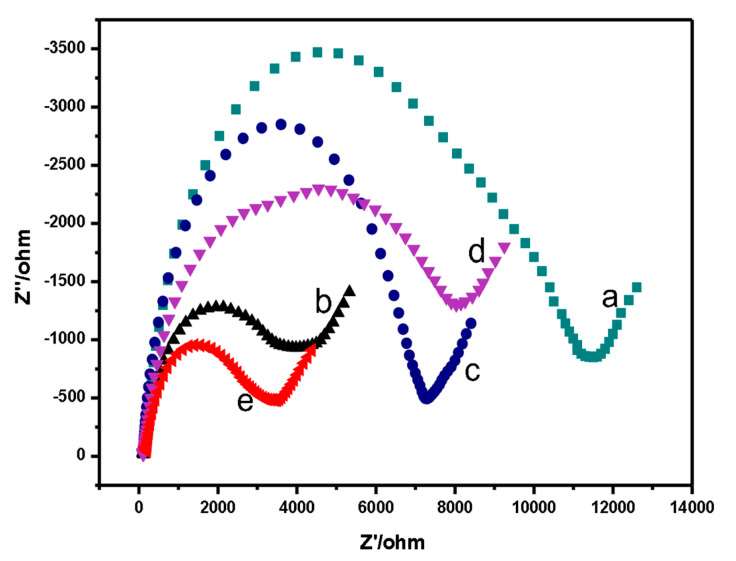
Electrochemical impedance spectroscopy (EIS) recorded in 5 mM of Fe(CN)_6_^3−/4−^ mixture (1:1) containing 0.1 M of KCl on (**a**) GCE, (**b**) GO/GCE, (**c**) ErGO/GCE, (**d**) UiO-66/ErGO/GCE, (**e**) UiO-66/GCE.

The Nyquist plots from the electrochemical impedance spectroscopy (EIS) test reflected the conductivities of different electrodes (Figure 2). The smaller semicircle diameter of the Nyquist curve meant smaller electron transfer impedance, and more exceptional electrical conductivity as well. As shown in Figure 2, electron transfer resistance of electrodes (R_et_) of GO/GCE increased after electrochemical deposition of GO on GCE. With the electrochemical reduction of GO/GCE, the conductivity of the electrode became higher [27], which explained the reason for the semicircle diameter of ErGO/GCE being smaller than that of GO/GCE. Moreover, compared to UiO-66/GCE (curve e), the decrease in R_et_ of UiO-66/ErGO/GCE (curve d) was due to the addition of carbon materials. Lower polarization resistance of UiO-66/ErGO/GCE compared with those of GCE and UiO-66/GCE indicated that the combination of UiO-66 and ErGO could be used as a good decorating material to promote the electron transfer between target and electrode.

### 2.2. Electrochemical Behavior of LU on UiO-66/ErGO/GCE

**Figure 3:**
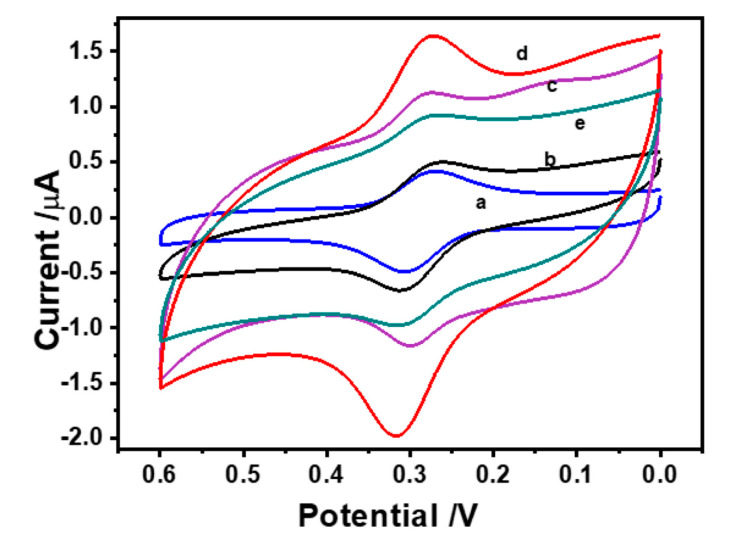
Cyclic voltammetries (CVs) of 0.5 μM of LU on (**a**) GCE, (**b**) GO/GCE, (**c**) ErGO/GCE, (**d**) UiO-66/ErGO/GCE, (**e**) UiO-66/GCE in PBS (pH = 6.0) at scan rate of 0.01 V s^−1^.

The electrochemical behavior of LU (0.5 μM) in phosphate buffer saline (PBS, pH = 6.0) on the different kinds of modified electrodes was evaluated by CV. Figure 3 indicated that five kinds of electrodes had varying degrees of redox reactions and different extents of signal of LU. The highest oxidation peak current (Ipa) was obtained on UiO-66/ErGO/GCE, which proved that the modifications of UiO-66 and ErGO made contributions to the signal enhancement of the electrode. As one of the modified materials, UiO-66 showed enrichment and molecular recognition abilities, which increased the binding between LU and the electrode surface [28]. However, the limitations of UiO-66 in conductivity was made up for by ErGO, which possessed high superficial area, high speed of electron transfer and strengthened electrochemical signals. It can be observed in Figure 3d that a simultaneous increase of the anodic and cathodic processes suggested an increase of superficial area on the surface of electrode caused by the modifications of ErGO and UiO-66. It could be assumed that the synergy of ErGO and UiO-66 led to the rise of Ipa by expanding the effective electrode area and accelerating the electrochemical reactions.

### 2.3. Optimization of Experimental Conditions

#### 2.3.1. Effect of pH

**Figure 4:**
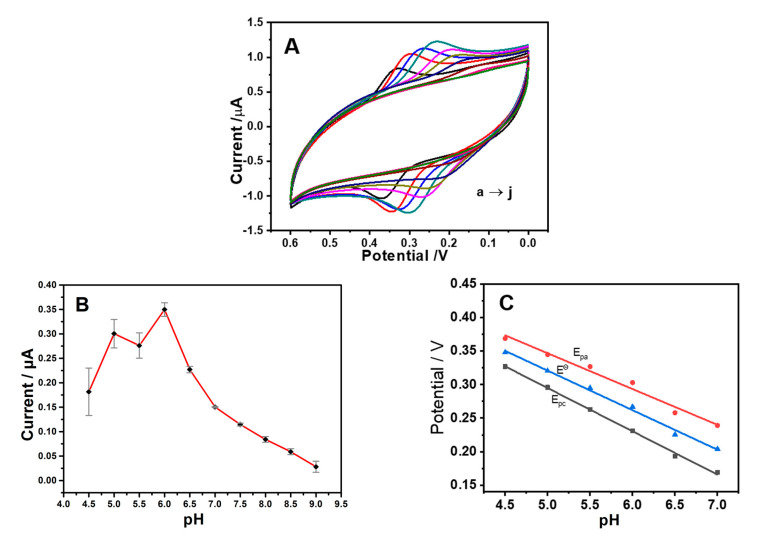
(**A**) CVs of 0.5 μM of LU on UiO-66/ErGO/GCE in various pH (a to j: 4.5 to 9.0) of supporting electrolyte solutions (scan rate of 0.05 V s^−1^). (**B**) Effect of pH on the Ipa of LU. (**C**) Effect of pH on the oxidation peak potential (Epa) of LU.

The electrochemical behavior of UiO-66/ErGO/GCE for LU with different pH was evaluated by CV (Figure 4A). As shown in Figure 4B, the Ipa was affected obviously when the acidity of the buffer solution changed. The Ipa reached the maximum value when the pH value was 6.0. Therefore, PBS under this condition (pH 6.0) was selected as the electrolyte solution for detecting LU. When the pH value was less than 6.0, the Ipa of the electrode fluctuated slightly. While the Ipa showed a downward trend when the pH value exceeded this value, it might be because the reduction of proton content made the reaction more and more difficult. On the other hand, the Epa gradually shifted to negative potential as the pH value increased, which also proved that protons were part of this reaction. As shown in Figure 4C, there was a linear relationship between Epc, Epa, E_Ɵ_ and pH shown as: Epc = −0.0646 pH + 0.6181 (R^2^ = 0.9979), Epa = −0.0534 pH + 0.6140 (R^2^ = 0.9764) and E_Ɵ_ = −0.0590 pH + 0.6161 (R^2^ = 0.9939). These trends were precisely the same as the theoretical value of the Nernst Equation. In other words, the number of transferred electrons and protons was exactly the same as those in the electro-oxidation reaction of LU, which followed a two-electron and two-proton mechanism [29].

#### 2.3.2. Effects of Accumulation Potential and Accumulation Time

**Figure 5:**
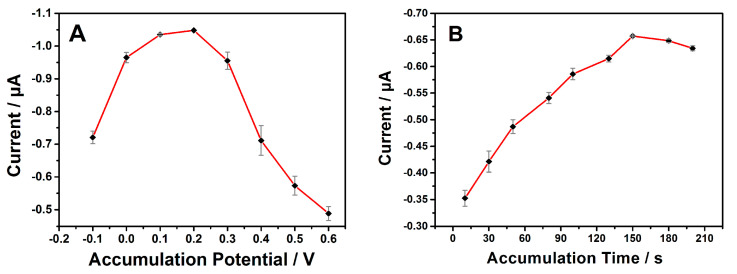
Relationship between peak current of LU and (**A**) accumulation potential and (**B**) accumulation time.

Accumulation was a method to increase the adsorption amount of LU on UiO-66/ErGO/GCE, so as to improve the sensitivity of the electrode. Therefore, it was necessary to explore the effects of the accumulation potential and accumulation time. As shown in Figure 5A, in the research range of −0.1 to 0.6 V, the peak current gradually increased and reached the maximum value under the condition of 0.2 V, after which it continued to decrease. Consequently, the accumulation potential was chosen at 0.2 V for the following analyses. The influence of accumulation time on the detection of LU was also conducted and shown in Figure 5B. The Ipa increased significantly in the range of 10 s to 150 s. However, it began to decrease when the accumulation time was longer than 150 s, which showed the impact on Ipa began to decrease. Finally, 150 s was selected as the optimum accumulation time for the following tests.

#### 2.3.3. Effect of Scan Rate

**Figure 6:**
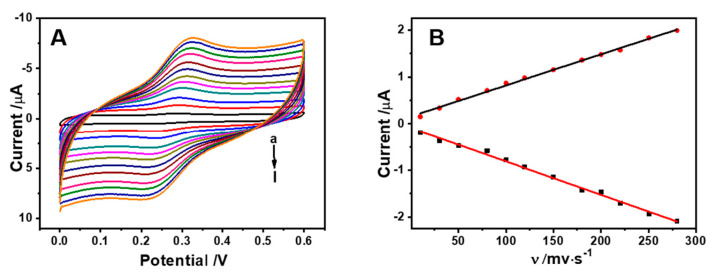
(**A**) CVs of UiO-66/ErGO/GCE in the presence of 0.5 μM of LU (pH = 6.0) at different scan rates ranging from a to l (0.01 to 0.275 V s^−1^). (**B**) Correlation between peak current vs. scan rate.

The effect of scan rate (0.01 to 0.275 V s^−1^) on the peak current of LU was performed in detecting 0.5 μM of LU in PBS (pH = 6.0). As shown in Figure 6, there were linear relationships between Ipa, Ipc and scan rate. The linear regression Equations were described as follows: Ipa = 6.6399 v + 0.1563 (R^2^ = 0.9968) and Ipc = −7.1546v − 0.0974 (R^2^ = 0.9949). The above results illustrated that the reaction of LU on the surface of UiO-66/ErGO/GCE was controlled by the adsorption procedure [30].

### 2.4. Quantitative Analysis of LU on UiO-66/ErGO/GCE

**Figure 7:**
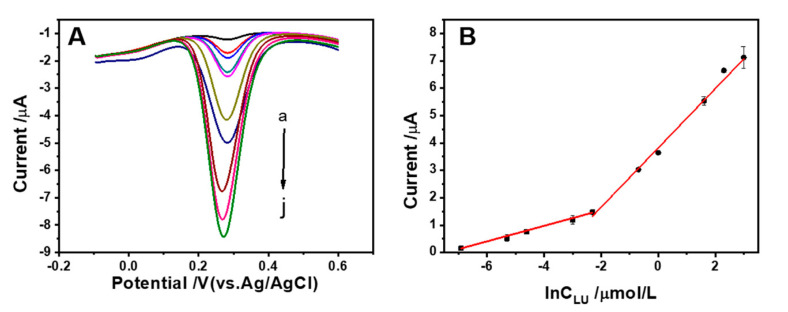
(**A**) Differential pulse voltammetry (DPV) of UiO-66/ErGO/GCE in 0.1 M PBS (pH = 6.0) with different LU concentrations from a to j (0.001, 0.005, 0.01, 0.05, 0.1, 0.5, 1, 5, 10 and 20 μM). (**B**) The corresponding calibration curves between Ipa and LU concentrations for UiO-66/ErGO/GCE.

Under the optimization conditions discussed above, differential pulse voltammetry (DPV) was used to complete the electrochemical responses for different concentrations of LU. It could be seen in Figure 7, the Ipa of LU gradually increased with continuously increasing concentration, and there were two different linear relationships at high and low concentrations between Ipa and lnC_LU_. At low concentrations of LU (0.001, 0.005, 0.01 and 0.05 μM): I = 0.2834lnC_LU_+ 2.0763 (R^2^ = 0.9936), while, at high concentrations of LU (0.05, 0.1, 0.5, 1, 5, 10 and 20 μM): I = 1.1038 lnC_LU_+ 3.8584 (R^2^ = 0.9940). The limit of detection was 0.75 nM of LU (S/N = 3), which was satisfied compared to reported sensors (Table 1). This kind of linearity was in accordance with report [31]. The performance of the electrochemical sensor for detecting LU proposed in this study was worthy of further development.

### 2.5. Anti-Interference Ability, Stability and Repeatability

**Figure 8:**
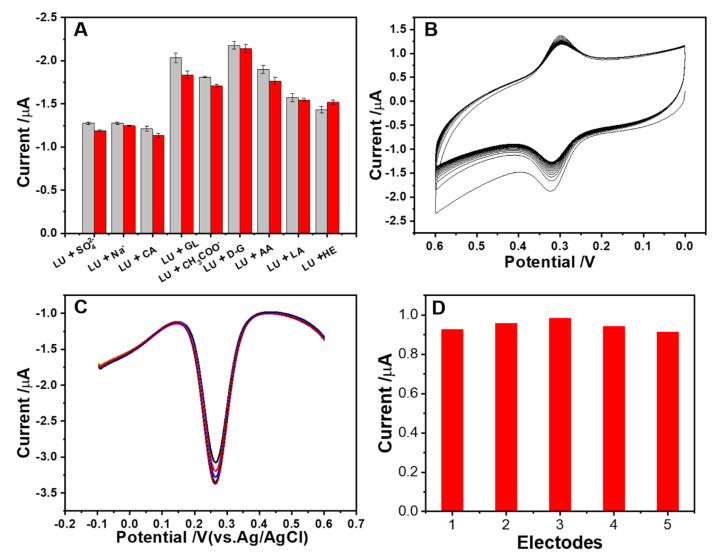
(**A)** Anti-interference ability: different types of interfering substances caused changes in the peak current of LU detected by DPV on UiO-66/ErGO/GCE. (**B**) Stability: CV of UiO-66/ErGO/GCE was repeated at 20 cycles in 0.5 μM of LU in PBS (pH 6.0), scan rate was 0.05 V s^−1^. (**C**) Repeatability: single modified electrode pair 0.5 μM of LU intermittently measured 8 times through DPV under the same conditions. (**D**) Reproducibility: repeated measurements of 0.5 μM of LU with multiple modified electrodes, changes in oxidation peak current.

In order to test the anti-interference ability of the electrode, a variety of interfering substances were added, such as inorganic ions, organic acids, sugars and other flavonoids, to explore the changes in the electrochemical response of UiO-66/ErGO/GCE for 0.5 μM of LU through DPV. As shown in Figure 8A, 50 times concentration of Na^+^, 10 times concentration of lauric acid (LA) and 10 times concentration of D-galactose (D–G) did not make any obvious interference (changes of analysis signal were less than 5%). For other interfering substances, which were 10 times the concentration of SO_4_^2−^, CH_3_COO^−^, caffeic acid (CA), ascorbic acid (AA), glucose (Glu) and hesperidin (He), the effects of them on currents were all less than 10%. These results showed that UiO-66/ErGO/GCE had good anti-interference ability for LU.

The stability test was conducted with a single electrode in 0.1 M PBS buffer solution, which was used in CV at a scan rate of 0.05 V s^−1^ and scan range from 0 to 0.6 V (Figure 8B). As the number of scan cycles increased, the redox peak current gradually decreased and finally reached stable after 20 cycles. For the repeatability test of UiO-66/ErGO/GCE, eight intermittent measurements were performed on 0.5 μM of LU (Figure 8C). The obtained relative standard deviation (RSD) value was 5.4%, which proved the repeatability of UiO-66/ErGO/GCE was acceptable. For the reproducibility test of UiO-66/ErGO/GCE, five electrodes prepared according to the same procedure were used to test LU in the same buffer solution. The result showed the RSD value was 2.9% (Figure 8D). Based on the properties of GO and UiO-66, the resulting modified electrode UiO-66/ErGO/GCE showed good anti-interference ability, stability, repeatability and reproducibility.

### 2.6. Detection of a Real Sample

In order to verify the application of UiO-66/ErGO/GCE for real samples, extracts of chrysanthemums and hawthorn were determined, which contained certain amounts of LU. In order to make the results more accurate, the standard addition method was used by adding three different concentrations of LU to samples under the optimal detection conditions [41]. As Table 2 showed, the recovery rates of test samples were 99.85–101.17%, and the RSD values were between 0.77 and 6.86%, which demonstrated the good performance and reliable results of UiO-66/ErGO/GCE for testing real samples.

## 3. Materials and Methods

### 3.1. Reagents and Apparatus

Luteolin, zirconium tetrachloride, terephthalic acid, hydrochloric acid, ethanol and N,N-dimethylformamide (DMF) were purchased from Shanghai Aladdin Biochem Technology Co., Ltd. (Shanghai, China). Graphene oxide was purchased from Nanjing XFNANO Materials Tech Co., Ltd. (Nanjing, China). Chrysanthemums (A), chrysanthemums (B) and hawthorns were bought at local supermarket in Xiangtan, China. Several kinds of polishing powders were bought from Shanghai Chenhua Instrument Company (Shanghai, China). Ultrapure water (18.25 MΩ cm) was obtained from a Millipore system (Millipore Corporation, Milford, MA, USA). All other chemicals were of analytical grade without further purification.

The electrochemical measurements were accomplished on a CHI660E electrochemical workstation (Shanghai Chenhua Instrument Company, Shanghai, China) using a standard three electrode system. The observations were completed by HSM 6610Lv scanning electron microscopy (JEOL, Tokyo, Japan) operated at 25 kV. A Bruker D8 advance X-ray diffractometer was used to study the crystalline structures of the powder samples. The Raman and FTIR spectra of samples were achieved using a Renishaw Invia Raman spectrometer and Thermo Scientific Nicolet iS10 spectrometer, respectively.

### 3.2. Preparation of UiO-66

UiO-66 was synthesized by a hydrothermal method [42]. Typically, 0.68 g of zirconium tetrachloride and 0.83 g of terephthalic acid were added to 60 mL of DMF and treated under ultrasonic for 1 h. After addition of 1 mL of hydrochloric acid (10%), the mixture was poured into a Teflon reactor and incubated at 120 °C for 16 h (DHG 9123A Oven, Jing Hong Laboratory Instrument Co., Ltd., Shanghai, China). After reaction and cooling of the reactor, the precipitate was separated using centrifugation and washed with DMF three times. Finally, the white powders of UiO-66 were collected and dried in a vacuum.

### 3.3. Preparation of Electrodeposition Solution of GO

The 20 mg of GO was weighed and placed in 10 mL of water and stirred with ultrasonic for about 10 min until a metallic black solution was formed and dissolved.

### 3.4. Preparation of UiO-66/ErGO/GCE

After polishing twice with 0.3 μm and 0.05 μm of alumina slurries, the GCE was ultrasonically washed in anhydrous ethanol and water for 5 min and dried in air. In order to form the ErGO modified GCE (ErGO/GCE), the fresh GCE was scanned for 20 sweep segments in the electrodeposition solution of GO by CV in the voltage range from −0.2 to −1.2 V with a scan rate of 0.1 V s^−1^. Then, the ErGO/GCE was scanned for 12 sweep segments in PBS (pH = 7.0) in the voltage range from −0.9 to 0 V with scan rate of 0.1 V s^−1^, washed with water and dried at room temperature. Finally, 5 μL of UiO-66 aqueous solution (2 mg mL^−1^) was dripped onto the surface of ErGO/GCE and dried in the air. The UiO-66 modified electrode was obtained and marked as UiO-66/ErGO/GCE.

### 3.5. Preparation of Samples

After being ground into powders (BJ-800A pulverizer, Baijie Electrical Appliances Co., Ltd., Huzhou, China), 0.40 g of chrysanthemums (A), 0.40 g of chrysanthemums (B) and 1.00 g of hawthorns were each weighed and soaked in 20 mL of hot water for 90 min under water bath (DZKWD4, Ever Bright Medical Treatment Instrument Co.,Ltd., Beijing, China). Then, the three samples were cooled to room temperature, filtrated to get clear liquid, and diluted to 100 times with PBS (pH = 6.0). The three sample solutions were stored at 4 °C for further detection.

### 3.6. Electrochemical Test

In this experiment, the CHI660E electrochemical workstation was used for research with CV from 0.0 to 0.6 V with scan rate of 0.01 V s^−1^ and DPV in the voltage range from −0.1 to 0.6 V with amplitude of 0.05 V and sampling width of 0.0167 s. The 0.1 M PBS was adopted as a buffer solution, and the measurements were conducted in nitrogen atmosphere. The standard three-electrode system used was composed of a platinum wire electrode, Ag/AgCl electrode and UiO-66/ErGO/GCE. The EIS test was achieved in an electrolyte solution, which contained 0.1 M of KCl and 5 mM of [Fe(CN)_6_]^4+^/[Fe(CN)_6_]^3+^. The impedance parameters were set as 0.01 V of amplitude and the frequency range was from 0.01 to 100 kHz.

## 4. Conclusions

In this paper, UiO-66/ErGO/GCE was fabricated for the detection of LU. Through the comparison of fabrication procedures, UiO-66/ErGO showed the highest oxidation peak current for LU. The factors affecting the electrochemical behavior of UiO-66/ErGO/GCE were evaluated and optimized including pH, accumulation potential, accumulation time and scan rate. Under the optimum conditions, UiO-66/ErGO/GCE showed satisfactory linearity, anti-interference ability, reproducibility and storage stability. It could provide at least eight stable detections for one UiO-66/ErGO/GCE, which guaranteed the practicability of the proposed sensor. The limit of detection of UiO-66/ErGO/GCE reached 0.75 nM of LU and was suitable for testing real samples. The proposed UiO-66/ErGO/GCE showed rapid electrochemical response, low consumption of materials and simple operation in detection of LU.

## Figures and Tables

**Table 1:** Comparison of the performance of UiO-66/ErGO/GCE with other reported LU sensors.

LU Sensors	Detection Limit (μM)	Linear Range (μM)	References
GCE	0.73	0.91–56.9	[32]
HAP-CNT/GCE	0.08	0.4–12	[33]
PDDA-G-CNTs/β-CD/GCE	0.02	0.05–60	[34]
MIP/MoS_2_/GN-CNTs/GCE	0.009	0.04–2.0	[35]
PCV/MWCNTs/GCE	0.005	0.020–70	[36]
MOF-801/MC/GCE	0.0029	0.02–10	[37]
Pt-BPC/CILE	0.0026	0.008–100	[38]
GQDs/GNPs/GCE	0.001	0.01–10	[39]
UiO-66/ErGO/GCE	0.00075	0.001–20	This work
Cu_x_Co_4-x_@NPCP composites	0.00008	0.0002–2.5	[4]
MWNTs/GCE	0.00006	0.0002–0.003	[29]
SH-β-CD-GNs/AuNPs/GCE	0.0000033	0.00001–10	[40]

Note: PDDA-G-CNTs/β-CD, graphene-carbon nanotubes hybrid/β-cyclodextrin composite film; MWNTs/GCE, multiwalled carbon nanotubes modified glassy carbon electrode; Cu_x_Co_4-x_@NPCP composites, MOFs derived CuCo coated nitrogen-doped porous carbon polyhedron; SH-β-CD-GNs/AuNPs, thio-β-cyclodextrin functionalized graphene/gold nanoparticles hybrids; HAP-CNT, alkaline phosphatase mediated carbon nanotube–hydroxyapatite nanocomposite; GQDs/GNPs/GCE, graphene quantum dots/gold nanoparticles nanocomposite modified electrode.

**Table 2:** Determination of LU concentration in real samples.

Samples	Added (μM)	Found (μM)	Recovery (%)	RSD (*n* = 3) (%)
Chrysanthemums (A)	0.00	0.1227	-	3.01
	0.5	0.6295	101.09	4.73
	1.0	1.1425	101.16	6.11
	1.5	1.6618	101.17	2.54
Chrysanthemums (B)	0.00	0.1382	-	1.89
	0.5	0.6394	100.19	2.31
	1.0	1.1382	99.89	0.77
	1.5	1.6444	100.38	2.41
Hawthorns	0.00	0.0992	-	3.21
	0.5	0.5983	99.85	6.86
	1.0	1.1075	100.84	5.37
	1.5	1.6197	100.76	1.38
